# Preparation, Characterization, and *In Vitro* Cytotoxicity Evaluation of a Novel Anti-Tuberculosis Reconstruction Implant

**DOI:** 10.1371/journal.pone.0094937

**Published:** 2014-04-16

**Authors:** JunFeng Dong, ShengMin Zhang, Jun Ma, HaoMing Liu, YingYing Du, YongHui Liu

**Affiliations:** 1 Advanced Biomaterials and Tissue Engineering Center, Huazhong University of Science and Technology, Wuhan, P.R. China; 2 Department of Biomedical Engineering, Huazhong University of Science and Technology, Wuhan, P.R. China; Glasgow University, United Kingdom

## Abstract

**Background:**

Reconstruction materials currently used in clinical for osteoarticular tuberculosis (TB) are unsatisfactory due to a variety of reasons. Rifampicin (RFP) is a well-known and highly effective first-line anti-tuberculosis (anti-TB) drug. Poly-DL-lactide (PDLLA) and nano-hydroxyapatite (nHA) are two promising materials that have been used both for orthopedic reconstruction and as carriers for drug release. In this study we report the development of a novel anti-TB implant for osteoarticular TB reconstruction using a combination of RFP, PDLLA and nHA.

**Methods:**

RFP, PDLLA and nHA were used as starting materials to produce a novel anti-TB activity implant by the solvent evaporation method. After manufacture, the implant was characterized and its biodegradation and drug release profile were tested. The *in vitro* cytotoxicity of the implant was also evaluated in pre-osteoblast MC3T3-E1 cells using multiple methodologies.

**Results:**

A RFP/PDLLA/nHA composite was successfully synthesized using the solvent evaporation method. The composite has a loose and porous structure with evenly distributed pores. The production process was steady and no chemical reaction occurred as proved by Fourier Transform Infrared Spectroscopy (FTIR) and X-Ray Diffraction (XRD). Meanwhile, the composite blocks degraded and released drug for at least 12 weeks. Evaluation of *in vitro* cytotoxicity in MC3T3-E1 cells verified that the synthesized composite blocks did not affect cell growth and proliferation.

**Conclusion:**

It is feasible to manufacture a novel bioactive anti-TB RFP/PDLLA/nHA composite by the solvent evaporation method. The composite blocks showed appropriate properties such as degradation, drug release and biosafety to MC3T3-E1 cells. In conclusion, the novel composite blocks may have great potential for clinical applications in repairing bone defects caused by osteoarticular TB.

## Introduction

Tuberculosis (TB) is a chronic, debilitating disease caused by Mycobacterium tuberculosis,and is the leading cause of death from a curable infectious disease [1]. One-third of the world's population (approximately 2 billion individuals) has contracted a TB infection, and 300 million people will be newly infected in the coming decade. With the increase in poverty, total global population and floating population over the past 20 years, as well as the emergence of multiple drug-resistant TB and increase in opportunistic infections due to the HIV/AIDS epidemic, controlling and treating TB have become much more serious and difficult than ever before [2]. Multiple organs and tissues of the body can be infected by TB, not only from newly acquired Mycobacterium tuberculosis infections, but also from older pulmonary TB foci. Osteoarticular TB is the most common site of extrapulmonary TB, accounting for 35–50% of extrapulmonary TB and approximately 3–5% of the total TB incidence. Nearly one-third of osteoarticular TB patients suffer from spinal TB [3]. During the past 10 years, with the overall increase in cases of TB, the incidence of both global spinal TB and drug-resistant spinal TB has significantly increased. Vertebral collapse and kyphosis occur during the latter period of spinal TB as a result of delayed treatment in the early stage, and nearly 10% of spinal TB patients develop paraplegia due to spinal cord or nerve compression. With the development of new surgical techniques and advanced biomedical biomaterials, the curative ratio of osteoarticular TB has greatly increased in the past twenty years. However, problems with effective reconstruction of bone defects caused by TB, treatment of drug-resistant strains and adverse side effects of existing anti-TB drugs remain to be solved [4]. The reconstruction implants used in osteoarticular TB include autogenous bone, allograft bone, and artificially synthesized biomaterials including calcium phosphate-based biocement materials, titanium mesh, and polymethylmethacrylate (PMMA). However, all of these materials have clinical limitations. For example, titanium mesh, which has been widely used in spinal TB in recent years, provides immediate stability and high mechanical strength; however, the high prosthesis subsidence ratio and the ease of bacterial adhesion to the titanium mesh surface has resulted in increased recurrence of spinal TB and a poor bone fusion ratio [5]. Currently, the recurrence rate of postoperative spinal TB remains as high as 13–26% [6]; therefore it is critically important to develop novel, ideal reconstruction materials for osteoarticular TB.

Poly(D,L)-lactic acid (PDLLA) and nano-hydroxyapatite (nHA) are two most promising orthopedic reconstruction materials due to their high biodegradability, non-toxic side effects and lack of immunogenicity [7], [8]. Additionally, they are both used as core materials in drug delivery systems (DDS) due to their impact on sustainable release and excellent drug delivery performance [9], [10]. Therefore, in the present study, we attempted to synthesize and characterize a novel anti-TB reconstruction implant comprised of these two materials in combination with Rifampicin (RFP) to address the difficulties in osteoarticular TB treatment. We have used PDLLA and nHA as a DDS for RFP, which is a widely used first-line anti-TB drug known to be highly effective against TB. In this preliminary screening, we also evaluated the *in vitro* cytotoxicity of this novel synthetic composite block using MC3T3-E1 pre-osteoblast cells.

## Materials and Methods

### 1 Materials

nHA powders (40–50 nm) was synthesized using the wet chemical method [11] at the Advanced Biomaterials and Tissue Engineering Center, Huazhong University of Science and Technology,Wuhan,China. PDLLA (M_w_: 100,000) was obtained from the Shandong Institute of Medical Instruments, Shandong, China. RFP (RFP, R3501), fluorescein diacetate (FDA, F7378) and propidium iodide (PI, P4170) were obtained from Sigma-Aldrich, St. Louis, MO, USA. The murine pre-osteoblast cell line MC3T3-E1 was obtained from the American Type Culture Collection, Rockville, MD, USA. The Cell Counting Kit-8 (CCK-8, ck04) was purchased from Dojindo Molecular Technologies, Inc, Gaithersburg, MD, USA. Dulbecco's Modified Eagle's Medium (DMEM), trypsin, *β*-Sodium glycerophosphate, 20% fetal bovine serum (FBS), penicillin, phosphate-buffered saline (PBS), streptomycin and hematoxylin-eosin (HE) staining kit were purchased from HuBei BIOS Biological Technology Co. LTD. (Wuhan, China). Sodium chloride, calcium nitrate, ammonia, ammonium biphosphate, dichloromethane and absolute ethyl alcohol were purchased from China National Medicines Co., LTD. (Shanghai, China). All chemicals and reagents were of an analytical or higher purity grade and were used as-received. CO_2_ of 99.5% purity was provided by a local air products company (Wuhan, China) and was used as-received.

### 2 Preparation and Characterization

200 mg RFP and 1.0 g PDLLA were first dissolved together in 6 mL dichloromethane at room temperature. This mixture (Liquid 1) was stirred at a speed of 1000 rpm until well-distributed. 200 mg nHA was uniformly dispersed in 2 mL absolute ethyl alcohol to be a suspension *via* sonication. This suspension was labelled as Liquid 2. These two liquids were mixed and stirred until uniform to be a suspension. The final suspension was labelled as Liquid 3. 2.5 g NaCl crystals with a diameter of 100–150 µm was initially used to fill a custom-made mold, then 600 µL Liquid 3 was added to completely immerse the NaCl crystals. The composite blocks were removed from the mold after 4-h in a drying oven at 37°C and were placed in culture dishes at 37°C overnight until completely dried. The blocks (Φ: 1.8 cm, δ: 0.6 cm) were then immersed in 500 mL distilled water for a minimum of 48 h in order to remove the NaCl crystals. The final RFP/PDLLA/nHA composites were lyophilized. The structure and morphology of the composite blocks were characterized using X-Ray Diffraction (XRD) (X'Pert PRO Diffractometer, PANalytical B.V, Holland), Fourier Transform Infrared Spectroscopy (FTIR, VERTEX 70, Bruker Corporation, Germany) and Environmental Scanning Electron Microscopy (ESEM, Quanta 200 ESEM, FEI Company, Holland) by conventional methods. The porosity was measured at 25°C using a pycnometer. The drug-loading and entrapment efficiency were determined using an Ultraviolet Spectrophotometer (TU-1810, Beijing Purkinje General Instrument Co., Ltd., Beijing, China).

### 3 In Vitro Degradation

Thirty-six replicate RFP/PDLLA/nHA composites were used for degradation experiment. The initial weight (W_o_) of each composite block was 50 mg. Each block was separately immersed in 350 mL PBS (pH 7.40) and stored in a constant temperature incubator at 37°C. Three blocks were taken out for testing once per week for total of 12 weeks. The weight loss ratio (WLR), pH, and ESEM evaluation were conducted on each block as described below. The control group was thirty-six porous PDLLA materials, which were produced as described above.

#### WLR

The removed blocks were immersed in distilled water for 24 h and lyophilized until a constant weight was reached. The residual weight (W_r_) was tested using an accurate Electronic Balance (FA1104N, Shanghai Precision Scientific Instrument Co., Ltd., Shanghai, China). The WLR was calculated using the following formula: WLR (%) = (W_o_-W_r_)/W_o_ * 100%.

#### pH Value

The pH of the blocks at different degradation time points were tested using a pH meter (PHS-3C, Shanghai Leici Instrument Factory, Shanghai, China).

#### ESEM

The lyophilized blocks were placed on the ESEM stage using a conductive adhesive. The morphology characteristics after degradation were observed after gold spraying.

### 4 Drug Release In Vitro

PBS with a pH of 7.4 was selected as the release medium in this study to mimic the *in vivo* environment. The concentration of released drug was measured using an Ultraviolet Spectrophotometer (TU-1810, Beijing Purkinje General Instrument Co., Ltd., Beijing, China). Three composite blocks (100 mg each) were suspended in 350 mL PBS, sealed in dialysis bags, and dialyzed against PBS at 37°C. The PBS was tested at different time points (12, 24, 48, 72 hr, and once a week thereafter for 12 weeks). The absorbance at 340 nm was measured and the cumulative release percentage (CRP) was calculated based on the standard curve.

### 5 In Vitro Cytotoxicity

In this study, we evaluated the *in vitro* cytocompatibility of the composites in both control and testing groups. In the control group, MC3T3-E1 pre-osteoblast cells were co-cultured with 10 mg porous PDLLA/nHA composite. In the testing group, MC3T3-E1 cells were co-cultured with 10 mg porous RFP/PDLLA/nHA composite. These two composites were both manufactured as described previously. For tests, they were plated in 48-well plates after sterilized with gamma radiation (25 kGy, Hubei Academy of Agricultural Sciences Radiation Center). 150 µL MC3T3-E1 cells suspension with a cell density of 3×10^5^/mL was seeded onto the surface of each block. Subsequently, 300 µL complete DMEM medium containing 10% FBS and 1% penicillin-streptomycin solution was added to completely submerge the whole blocks. Then the blocks were cultured in a 37°C, 5% CO_2_ incubator. The culture medium was replaced every 2–3 days and the cytotoxicity was evaluated using the assays described below.

#### Live/Dead Staining

After a 7-day co-culture, the live/dead cell staining was performed to evaluate the survival and growth of MC3T3-E1 cells in composite blocks. The fluorescent dyes used for determination of live and dead cells were fluorescein diacetate (FDA) and propidium iodide (PI), respectively. FDA and PI were added at a final concentration of 1 mg/mL and 20 µg/mL, respectively and were incubated for 30 min. Then the fluorescence of FDA and PI was measured at excitation wavelengths of 494 nm and 536 nm, respectively using a confocal laser scanning microscope (FV500, OLYMPUS Corporation, Japan). The live cells ratio (LCR) was analyzed by MATLAB (version 7.9.0.529) (MathWorks Corporation, Massachusetts, USA).

#### HE Staining

After a co-culture for 7 days, the composite blocks were stained with hematoxylin-eosin to evaluate the growth of MC3T3-E1 cells. The blocks were removed and washed with PBS to remove dead cells. Then the blocks were fixed in 4% paraformaldehyde for 12 h, dehydrated in a gradient of ethanols (50%, 75%, 85%, 95% and absolute ethanol) and embedded in paraffin. The paraffin-embedded blocks were horizontally cut into serial sections with a thickness of 5 µm by using a microtome. Sections were then deparaffinized in xylene-substitute transparent agent, rehydrated through a descending ethanol series and stained with haematoxylin-eosin. Then histological examination was carried out by using a light microscopy (DMIL-PH1, Leica Microsystems Ltd., Wetzlar, Germany).

#### Proliferation Rate

In order to evaluate the proliferation of MC3T3-E1 cells that had been co-cultured with porous composite blocks for 1, 3, 5, or 7 days, the CCK-8 kit was used according to manufacturer's instructions. The blocks were removed after the inner cells were digested with trypsin for 3 min. The optical density (OD) values of the remaining cell suspensions were measured at 450 nm using a plate reader (FLUOstar Omega, BMG Corporation, Germany) immediately after the co-culture.

### 6 Statistical Analysis

The data were analyzed using SPSS 19.0 software. The data within a given group or between groups were compared using One-way analysis of variance (ANOVA). The significant difference was defined as *p<0.05*.

## Results

### 1 Characteristics

**Figure 1:**
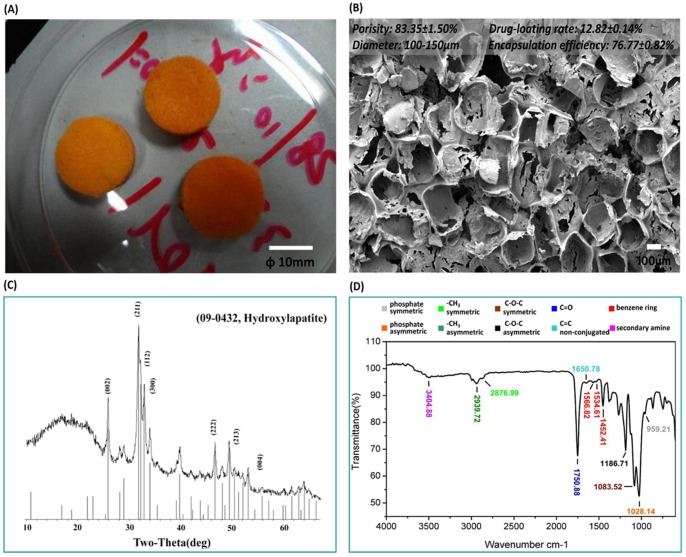
Characteristics of synthesized RFP/PDLLA/nHA composite. A:At the macroscopic level, RFP/PDLLA/nHA composite resembled a soft, porous orange sponge. [Scale bar, 10 mm]. B: ESEM micrograph showing even distribution of pores. The majority of pore walls and bottoms were smooth, while the remainder were irregularly cracked. [Scale bar, 100 µm]. C: XRD spectra; the diffraction peaks of inorganic constituents in the composite blocks conformed to the characteristic peaks of standard card No. 09-432 of JCPDS at 25.8°, 31.7° and 32.2°, indicating the presence of nHA. D: FTIR spectrum showing both RFP and PDLLA in the absence of newly produced contaminants derived from cross-linking or coupling reactions during the synthesis process.

The RFP/PDLLA/nHA composite had an orange color. It was soft, sponge-like, and porous (*Figure 1:* *A*). The pores were evenly distributed, and most walls and bottoms of the pores were smooth, while the remainder exhibited irregular cracks (*Figure 1:* *B*). No visible nHA crystals attaching with the walls and bottoms were detected by ESEM. The XRD spectrum indicated the presence of diffraction peaks of inorganic constituents in the composite blocks conformed to the characteristic peaks of standard card No. 09-432 of JCPDS at 25.8°, 31.7° and 32.2° (*Figure 1:* *C*). Taken together, the results confirmed the presence of nHA.

FTIR was used to detect the varieties of chemical groups in fabrication process. The absorption of phosphate symmetric stretching vibration at 959.21 cm^−1^ and phosphate asymmetric stretching vibration at 1028.14 cm^−1^ indicated the existence of HA in the composite blocks (*Figure 1:* *D*), The 2939.72 cm^−1^ position indicated a methyl asymmetric stretching vibration absorption peak and the 2876.99 cm^−1^ position indicated absorption peaks of methyl symmetric stretching vibration. The 1750.88 cm^−1^ position showed the ester carbonyl stretching vibration absorption peaks of C = O and 1186 cm^−1^ and 1083 cm^−1^ showed ester C-O-C asymmetric and symmetric stretching vibration absorption peaks, respectively. Together, these FTIR spectra identified the existence of PDLLA. The stretching vibration absorption peak of non-conjugated double bond C = C was present at 1650.78 cm^−1^. We observed the stretching vibration absorption peaks of the benzene ring carbon skeleton at 1452.41 cm^−1^, 1534.61 cm^−1^ and 1566.82 cm^−1^. A secondary amine stretching vibration peak was identified at 3404.88 cm^−1^, which was indicative of RFP. More importantly, there were no newly produced cross-linking, coupling or novel contaminants during the synthetic process.

The porosity of prepared RFP/PDLLA/nHA composite was 83.35±1.50%. The equation of the standard curve of RFP in dimethylformamide at 340 nm was calculated as Abs = −0.0959+0.6683*C (*µg/mL, R^2^ = 0.9795, n = 5*). The average drug loading rate and effective encapsulation efficiency were 12.82±0.14% and 76.77±0.82%, respectively.

### 2 In Vitro Degradation

#### WLR

**Figure 2:**
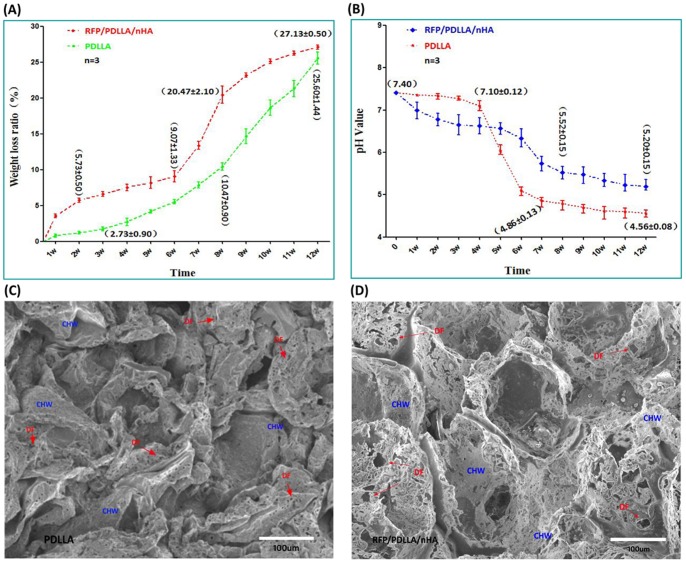
Degradation performance of RFP/PDLLA/nHA composite. A: Weight loss ratio in PBS; B: Variation in pH Values; C, D: ESEM micrograph showing degradation of two kinds of composites after 12 w in PBS; DF: degradation foraminulum, CHW: collapsed hole-wall. Each curve represents the mean ± standard deviation of 3 independent experiments performed in triplicate in A and B.

The WLR of pure porous PDLLA in PBS was initially low, then gradually increased to 2.73±0.90% during the first 4 w, followed by a quickened degradation phase, in which WLR rapidly increased to 10.47±0.90% by 8 w and 25.6±1.44% at 12 w, indicating that approximately ¼ of PDLLA had degraded within 3 months. In contrast, the decrease in weight of composite blocks occurred extremely rapidly, reaching 5.73±0.50% by 2 w. After the initial rapid degradation phase, the blocks underwent a relatively slow degradation, in which the WLR ascended slowly and steadily. Concomitant with the rapid degradation, the WLR increased significantly after 6 w, reaching 20.47±2.10% by 8 w and achieving a maximum WLR of 27.13±0.50% at 12 w. The WLR of blocks remained higher than porous PDLLA (*Figure 2:* *A*).

#### pH Value

The pH value of PDLLA group descended slowly during the first 4 w from an initial value of 7.40 to 7.10±0.12 by 4 w. Between 4 w and 7 w the pH descended rapidly, followed by another slow and steady decline. During the last 4 w, the pH value remained steady at approximately 4.5 without obvious alterations. The composite blocks exhibited a rapid pH decrease during the first 3 w, particularly during the first 2 w. The absolute pH value was lower than in the PDLLA group; however, it decreased slowly from 3 w to 5 w, and more rapidly after 6 w, descending to 5.52±0.15 by 8 w. Thereafter, the average pH value varied slowly, remaining above 5.0, and reached a final average pH of 5.19 by the end of the 12-week testing period. Despite the gradual decrease in pH of the RFP/PDLLA/nHA composite, the pH was consistently and significantly higher compared to control after week 5 (*Figure 2:* *B*).

#### ESEM

ESEM was performed to evaluate the morphological changes after a prolonged incubation of the composite blocks in PBS. Both types of porous materials exhibited uneven surfaces. They were both, to a certain extent, collapsed and contained vesicular structures after a degradation period of 12 w (*Figure 2:* *C, D*). Many irregular insect bite-like degradation foraminulum (DF) appeared on the wall of hole. The pure PDLLA collapsed to a greater extent than RFP/PDLLA/nHA composite; however, the DF of the latter was much more widespread and larger than that of the former, suggesting that the composite blocks degraded faster than the pure PDLLA by 12 w (*Figure 2:* *D*). These results were in accordance with the previously described differences in WLR between PDLLA and the blocks.

### 3 Drug Release Profile

**Figure 3:**
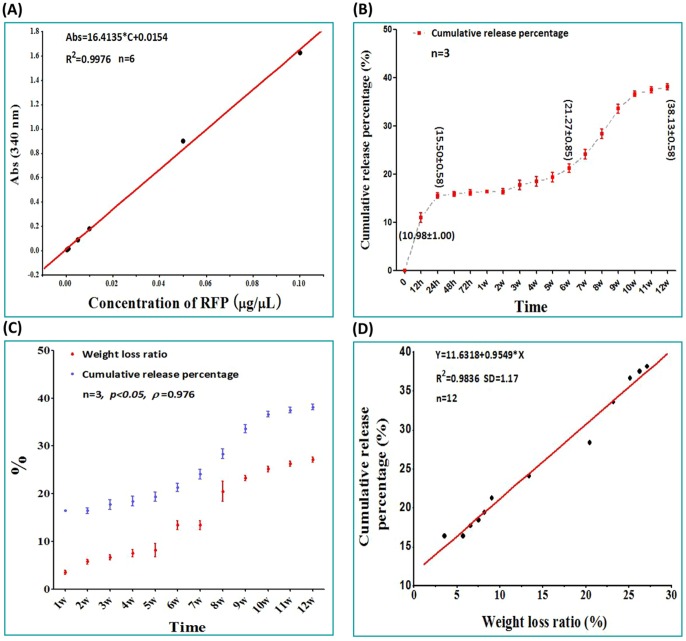
Drug release curve of RFP/PDLLA/nHA composite. A: Standard curve of RFP in PBS. B: Drug release curve of RFP in PBS. C: The relationship between drug release and degradation. D. The curve of best fit of the weight loss ratio and cumulative release percentage. Each curve represents the mean ± standard deviation of 3 independent experiments performed in triplicate in B and C.

The equation of the standard curve of RFP in PBS at 340 nm was calculated as Abs = 16.4135*C+0.0154 (*µg/µL, R^2^ = 0.9976, n = 6*) (*Figure 3:* *A*). Concomitant with slow release and dissolution of RFP, the release medium (PBS) turned from colorless to reddish-orange, gradually intensifying in color during the first 24 h. RFP/PDLLA/nHA composites exhibited an irregular burst release phase in the first 24 h (*Figure 3:* *B*). The CRP at 12 h and 24 h was 10.98±1.00% and 15.50±0.58%, respectively, during which time the PBS had not yet become orange in color. During the slow and steady release phase from 24 h to 6 w, there was a minimal variation in color, with a drug release rate of only 21.27±0.85% within 6 w. However, after 6 w there was a sudden acceleration of drug release. At 9 w, the color of the release medium had darkened slightly, becoming reddish orange by 9 w, after which 38.13±0.58% of the total drug was slowly and steadily released until 12 w, suggesting that the composite blocks possessed good sustained-release effects *in vitro* for at least 3 months.

The CRP of drug was significantly correlated with the rate of material degradation (*p<0.05*, *ρ = 0.976*) (*Figure 3:* *C*). The significant increase in CRP was accompanied by increasing material degradation, and the relationship between WLR and CRP was calculated as fitting the following curve: Y = 11.6318+0.9549*X (*R^2^ = 0.9836, SD = 1.17, n = 12*) (*Figure 3:* *D*).

### 4 In Vitro Cytotoxicity

#### Live/Dead Staining

**Figure 4:**
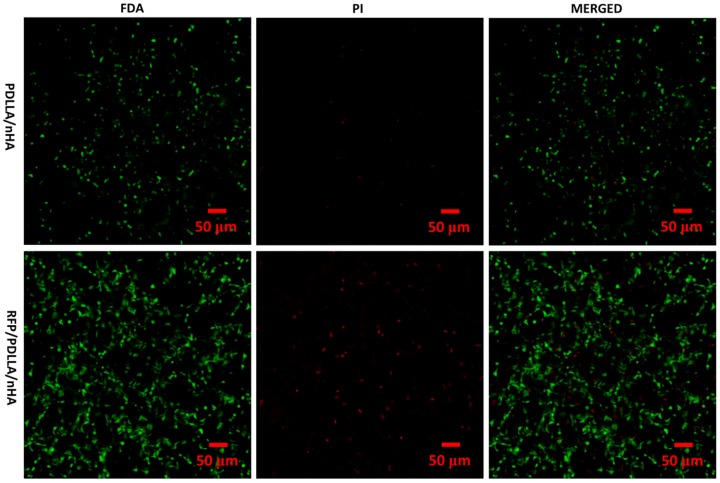
Confocal microscopy of co-cultured MC3T3-E1 cells. After 7 days in co-culture, imaging of FDA(green)/PI(red) double staining showed that MC3T3-E1 cells grew well within the two composites (PDLLA/nHA, RFP/PDLLA/nHA). A small number of dead cells (red) were scattered irregularly among the numerous live cells (green) in the two composites. [Scale bar, 50 µm].

MC3T3-E1 pre-osteoblast cells were cultured in porous RFP/PDLLA/nHA composites for 7 days. FDA/PI double staining was performed to analyze the live and dead cells by using an Olympus V500 confocal microscope (*Figure 4*). FDA is a non-fluorescent compound that is internalized by live cells and can be converted to fluorescein, which exhibits green fluorescence at an excitation wavelength of 494 nm. In contrast, PI can only penetrate damaged membranes of fixed or dead cells, and the orange fluorescence of PI can be visualized at an excitation wavelength of 536 nm. Thus the live/dead cell ratio can be determined by FDA/PI double staining. As shown in images (*Figure 4*), most of cells in the two composites were viable and healthy. The few PI-stained dead cells were scattered irregularly among the live cells in both composites and were clearly outnumbered by live cells. The live cells ratio (LCR) of two composite blocks were both more than 90% analyzed by MATLAB (version 7.9.0.529) (*Table 1*).

**Table 1:** Live cells ratio of MC3T3-E1 cells in two composites.

GROUP	FDA (G channel)	PI (R channel)
**PDLLA/nHA**	1078	41
**RFP/PDLLA/nHA**	3078	324
**Live cells ratio**	98.69%	90.48%

Analyzed by MATLAB (version 7.9.0.529); Binaryzation threshold: 20, Pixel threshold: 12.

#### HE Staining

**Figure 5:**
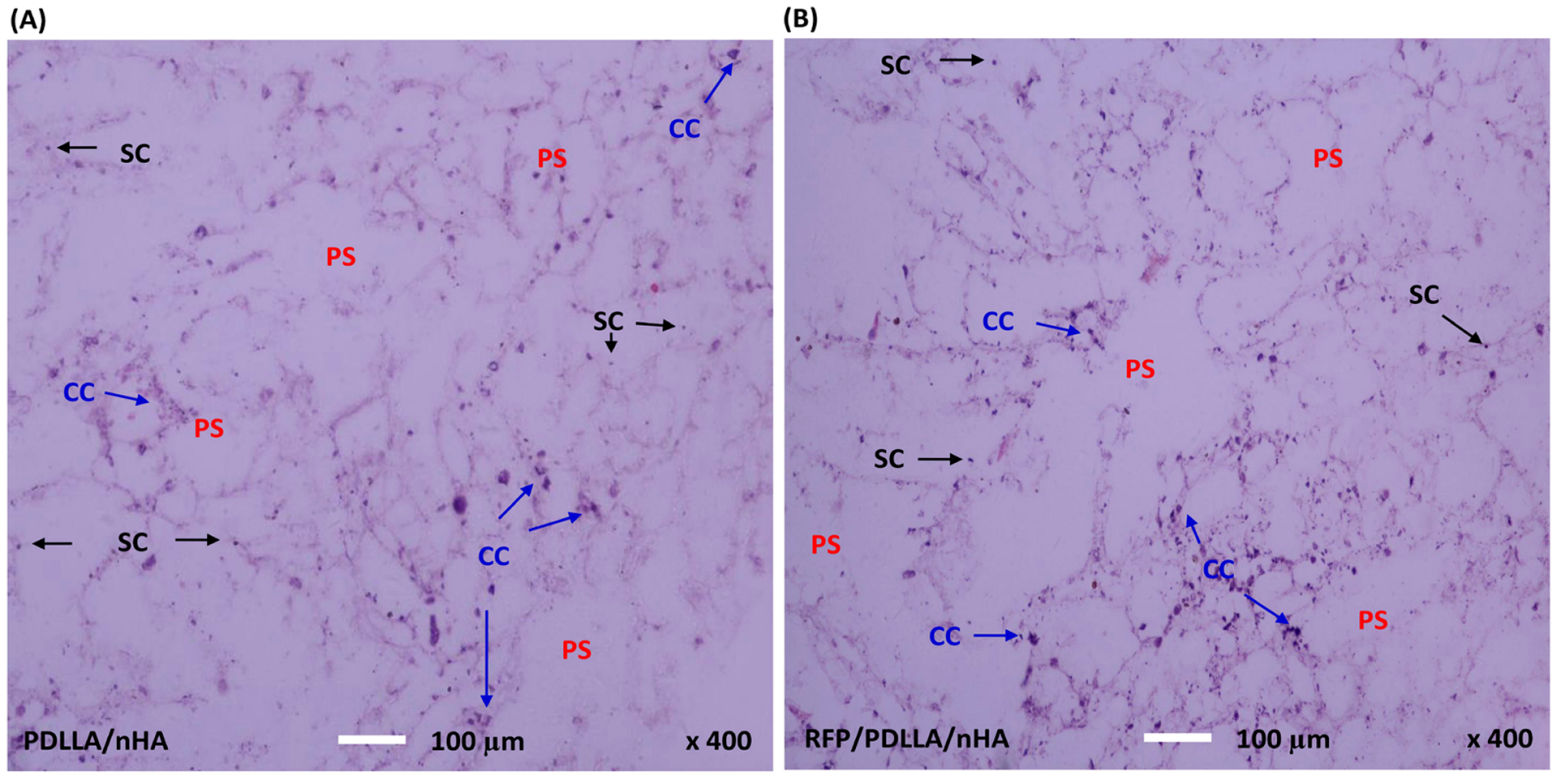
Histological evaluation of MC3T3-E1 cells. After 7 days of co-culture with MC3T3-E1 cells, the horizontal composites sections with a thickness of 5 µm were stained with hematoxylin-eosin and observed by a Leica light microscopy (400x magnifications). MC3T3-E1 cells proliferated within the two kinds of composites, and formed numerous cell colonies (CC). Cells stably adhered to the walls of the inner holes and were randomly distributed along the walls. A: PDLLA/nHA, B: RFP/PDLLA/nHA; PS: porous structure;SC: single cell. [Scale bar, 100 µm].

After a 7-day co-culture, the HE staining showed that MC3T3-E1 pre-osteoblasts could survive and maintain growth in the two kinds of composites (*Figure 5*), indicating that both composites exhibited good biocompatibility. HE-stained images of the two composites clearly demonstrated the inner porous structure, in which the cells had stably adhered to the walls of the inner holes, with the majority of cells scattered and growing along the walls. Additionally, many cell colonies formed in both composites, confirming the excellent cytocompatibility of the two composites.

#### Proliferation Rate

**Figure 6:**
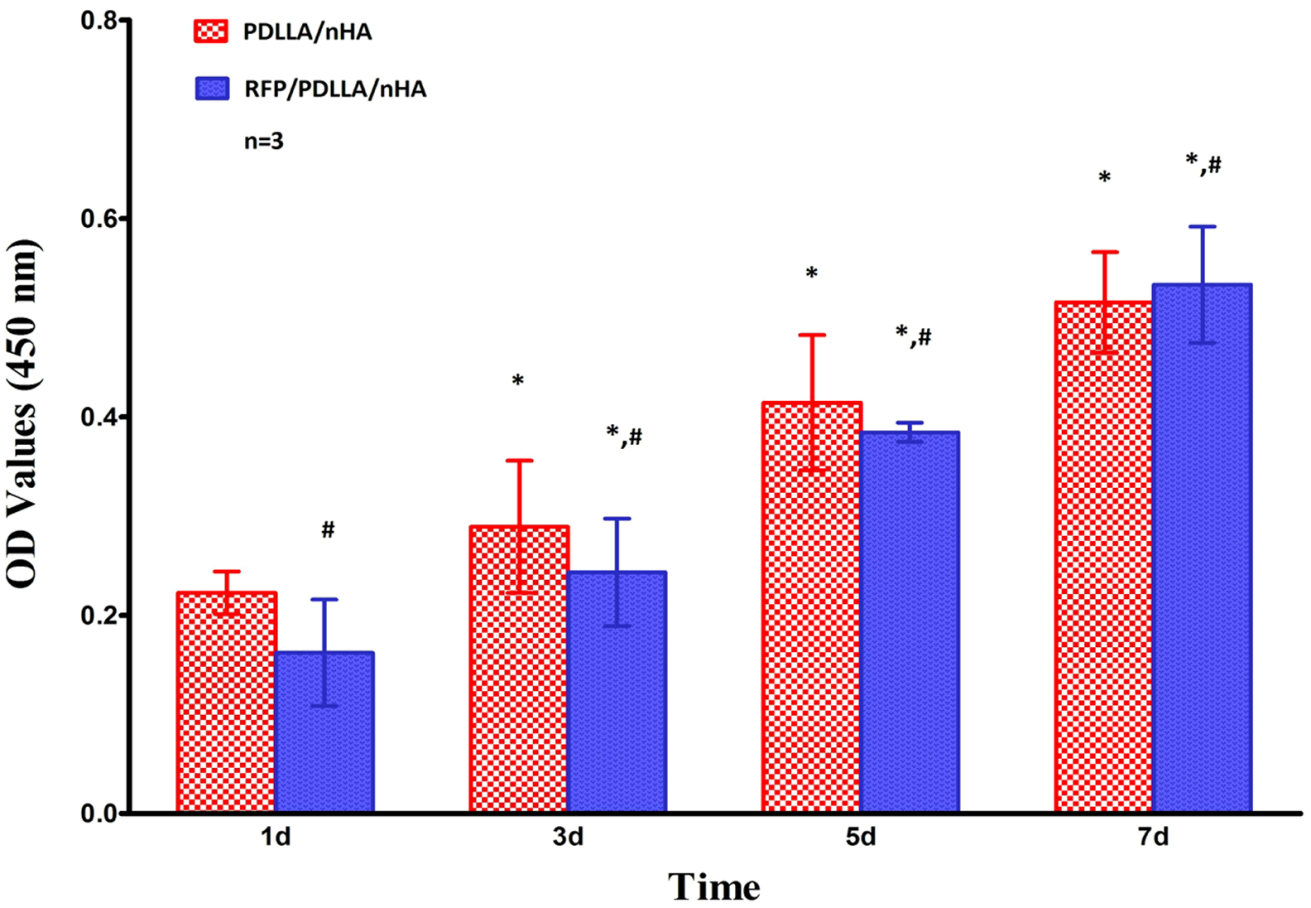
Proliferation of MC3T3-E1 cells. Each bar represents the mean ± standard deviation of 3 independent experiments performed in triplicate. *, *p<0.05* compared to the former testing time in the same group; ^#^, no significant differences were found when compared with the PDLLA/nHA group by one-way ANOVA (*p>0.05*).

The OD values of CCK-8 obtained from mouse pre-osteoblast MC3T3-E1 cells after co-culture within the two porous composites (porous PDLLA/nHA composite,porous RFP/PDLLA/nHA composite) are shown in *Table 2*. All OD values in the two groups increased with prolonged time in culture, which indicated ongoing proliferation of the mouse pre-osteoblast cells MC3T3-E1 (*p<0.05*) (*Figure 6*). Meanwhile, there were no significant differences between the OD values of the two groups (*p>0.05*), suggesting that the two composite blocks have no obvious cytotoxicity or adverse effects on cell proliferation.

**Table 2:** The OD values of MC3T3-E1 cells at 450 nm in two groups (n = 3, X±S).

GROUP	TIME (day)			
	D1	D3	D5	D7
**PDLLA/nHA**	0.2226±0.0214	0.2892±0.0666*	0.4143±0.0681*	0.5154±0.0508*
**RFP/PDLLA/nHA**	0.1621±0.0535#	0.2432±0.0541* ^,^ #	0.3844±0.0097* ^,^ #	0.5333±0.0586* ^,^ #

*, p<0.05;Compared with the former testing time using One Way ANOVA.

# *, p>0.05*;Compared with PDLLA/nHA group using One Way ANOVA.

## Discussion

PDLLA and nHA are two good drug carriers for osteomyelitis, however, the usage of them in osteoarticular TB remains to be further explored [12]. In this study, PDLLA and nHA were used not only as reconstructing materials but also vehicles for drug delivery. There are numerous preparation processes for HA/polymer composites [13], including the solvent evaporation method, hot-melt method, biomimetic method, electrospinning method, plasma spraying, in-situ polymerization process, coprecipitation method, liquid phase adsorption method and thermally-induced phase separation (TIPS). Among these processes, the solvent evaporation method has been widely adopted for its cost-effectiveness and ease of use. More importantly, it does not introduce ion impurities, and scaffolds fabricated in this way maintain favorable mechanical properties [14]. RFP is a first-line anti-TB drug recommended by WTO. The minimum inhibitory concentration (MIC) for *M. tuberculosis* is only 5 µg/mL [15]. It enabled to approach 10 times the MIC in serum after administration of the commonly used oral dose and speedily distribute in certain organs or tissues, especially the tuberculosis foci with a high density drug. Unfortunately it was proved that the concentration of RFP is extremely lower in bone tissues [16]. So it is very difficult to reach an effective RFP content in osteoarticular TB. Usage of high doses of RFP in osteoarticular TB treatment might lead to more serious complications to liver or kidney [17]. By contrast, it was easy for the other anti-TB drugs such as INH, streptomycin, and ethambutol to assemble in bone tissue [18]. Thus, RFP was set as the core drug in this study. However, oil-soluble RFP exhibited poor heat-resistance, with a melting point of only 183–188°C. In order to avoid degeneration and decomposition of RFP during preparations requiring higher temperatures, the solvent evaporation method was adopted.

The mechanical properties of the initial PDLLA/HA composite with high HA content were comparable to that of human bone [19]. However, aside from elastic modulus, properties including young's modulus, bending strength, and strain energy release rate decreased quickly concomitant with degradation. Specially, the elastic modulus was mainly controlled by HA content. The composites possessed higher elastic modulus when the HA content ranged from 20% to 60% [20]. However, mechanical properties should not be the only consideration in designing anti-TB bone implant. The composites composed of 5–10% nHA in wt.% with PDLLA illustrated excellent mechanical property and degradation performance when fabricated by melt-spinning [21]. Based on the results of our preliminary experiments, a ratio of 10% for nHA/PDLLA was chosen by weight in this study.

The structure is beneficial for new bone ingrowth when the pore size of the scaffold exceeds 50 µm and for bone conduction when the size of scaffold is larger than 80–100 µm [22]. However, increases in the degree of porosity and pore size were accompanied by a decline in mechanical properties. The pore size and degree of porosity of the scaffolds can also effectively control the drug release behavior of PDLLA/nHA scaffolds [23]. Pore sizes in the range of 100–400 µm are considered the most beneficial for adhesion and proliferation of osteoblast cells as they provide suitable pressure and tension for cellular mechanical stimulatory receptors [24]. The prepared RFP/PDLLA/nHA composite resembled porous orange foam, containing uniformly arranged pores ranging from 100 to 150 µm in diameter. Most walls and bottoms of the pores were smooth, while the remainder exhibited irregular cracks. The mechanical property, degradation and viscosity of polymers are associated with its molecular weight [25]. The cracks of walls might be related to the relatively low molecular weight of PDLLA. XRD testified the existence of nHA. However, they were not found in the pores. Maybe the overwhelming majority of nHA were inextenso distributed in the bottoms and walls of the pores. The structural characteristic offered greater surface area for cell adhesion, growth and distribution, and was also conducive to material vascularization. Meanwhile, the connected porous structure is crucial for the transfer and exchange of nutrients and oxygen.

In a recent study on osteomyelitis [26], PDLLA/HA/vancomycin composites showed excellent, sustained antibacterial effects on methicillin-resistant Staphylococcus aureus (MRSA). The PDLLA scaffold containing 28 µm HA fiber coating steadily released up to 23% vancomycin after 28 days. Drug release analysis on dexamethasone/PDLLA/nHA composites showed that dexamethasone release profiles were comprised of an initial burst phase within the first 10 h, followed by a slow release phase in next 790 h [27]. The initial burst stage was a consequence of free drug in close proximity to the surface of the drug-coated layer. The release rate of the scaffolds during the first 30 h highly correlated with the concentration of nHA and was attributable to the incorporation of hydrophilic nHA, which improved the drug loading capacity of the scaffolds. In our study, we also described an early burst of drug release in RFP/PDLLA/nHA composite, after which the release rate gradually stabilized, and remained stable for at least 12 w. The close linear relationship between drug release and degradation indicated that RFP was dispersedly distributed, which enabled the maintenance of a high drug concentration and continuous exertion of bactericidal effects. Large numbers of DF formed during the period of gradual drug release and material degradation. These structures are highly accessible by pseudopodia, thus promoting cell adhesion, and are also beneficial for intercellular transfer of nutrients or physiological signals. It indicated that the addition of nHA greatly improved the bone repair capability, but also significantly adjusted the degradation rate of PDLLA, which was more conducive to drug release.

Because of its gating-barrier effect, nHA was often mixed with polymers to adjust the degradation rate [28]. However, the WLR of RFP/PDLLA/nHA composite was faster than porous PDLLA. It is related to the drug release accompanied with the degradation. Especially the weight of the composite blocks lost fast during the initial stage. This phenomenon may be related to an early burst of drug release. Meanwhile, a newly research showed that the addition of nHA enabled to increase the water absorption ability and hydrophilicity of scaffolds [29], which would be beneficial for the permeation of degradation media and the ester chain hydrolysis of PDLLA. In a previous study [30], two kinds of PLLA/HA composite rods and unfilled PLLA rods were implanted in the subcutis and in the medullary cavities of rabbits. The results showed that the molecular weight of the PLLA/HA composites began to decrease at 2 weeks and decreased at a significantly faster rate than the unfilled PLLA rods. In unfilled PLLA rods, the molecular weight began to decrease at 4 weeks. The other reason might be that RFP molecules interfered with the intermolecular crosslinking of PDLLA. It was proved that the existence of high content of RFP might influence the bending instability and the formation of fabricating poly (l-lactic acid) (PLLA) fibers by electrospinning [31]. However, the concrete mechanism remained to be explored in the future. In addition, nHA played a significant role in modulating the changes in pH during the process of PDLLA degradation, which was more advantageous to osteoblast cell growth [32].

Consistent with its inhibitory effects on DNA synthesis, RFP exerts deleterious effects on osteoblast growth and proliferation at concentrations exceeding 50 µg/mL. This inhibition of growth and proliferation was particularly significant at concentrations up to 250 µg/mL [33], [34]. Sustained-release preparation is one of the most common types of method to avoid the negative affection of RFP. In a recent research [35], a dual drug-release coating was sprayed on the surface of polypropylene (PP) mesh using an airbrush spray system to prevent mesh-related infection. This coating was composed of three layers containing ofloxacin and RFP dispersed in a degradable polymer reservoir made up of [poly(ε-caprolactone) (PCL) and poly(DL-lactic acid) (PLA)]. The *in vitro* experiment testified that the coating was safe and effective due to its excellent drug release performance, although it exerted a short cytotoxic effect to the L-929 fibroblasts. In a previous *in vivo* study [36], a new vascular graft, preloaded with RFP and tobramycin was used in infrarenal aortic bypass for 12 dogs. The healing, toxicity, and the antibiotic delivery were evaluated after implanted for 7, 14 and 21 days. Histological examination showed that the healing of a novel RFP/tobramycin-bonded gelatin-sealed grafts was similar to that of commercial grafts, without any signs of toxicity. The gelatin-sealed RFP could even be detected for 21 days. In this study, multiple observations on adhesion, ingrowth, and proliferation of co-cultured MC3T3-E1 cells were conducted to evaluate cytotoxicity. In virtue of its excellent biosafety and osteoinductive, porous PDLLA/nHA scaffolds had been successfully applied to repair the rabbit femoral condyles defect [37], [38]. Therefore, porous PDLLA/nHA composite was set as control group. The results showed RFP/PDLLA/nHA composite possessed similar cell affinity, cell adaptation and no obvious inhibitory effects on cell growth to porous PDLLA/nHA composite, indicating that the addition of RFP was non-cytotoxic to pre-osteoblasts while maintaining its potent anti-TB effects. This might due to the addition of nHA, which reduced or partial eliminated RFP-induced inhibition of cell growth. The underlying mechanism between the influence of nHA and RFP on MC3T3-E1 cells will be further explored. In addition, it may be related to its excellent drug release profile, which ensured a steady lower concentration of RFP.

## Conclusion

This paper describes the conceptual design, preparation, characterization and *in vitro* cytotoxicity and adaptability evaluation of a novel degradable porous anti-TB reconstruction implant. We have developed a biocompatible and biodegradable composite blocks using PDLLA and HA as a DDS for RFP. The starting materials are all readily available and are approved for use by the Food and Drug Administration. The synthetic process is easily duplicated and widespread in practice. The RFP encapsulated by degradable PDLLA and HA exhibits controlled drug release concomitant with degradation, which demonstrates great potential for anti-TB effects. The highly porous structure may be advantageous for cell growth on inner surfaces of the materials, which is a fundamental and critical step in tissue repair. This novel implant comprised of RFP, PDLLA and HA may possess excellent osteogenic ability, making it an adequate bone substitute material.

Although the composite blocks we synthesized have great potential applications in bone repair, drug delivery and anti-TB effects, the true efficacy must be further investigated *in vitro* and in validated animal models in the future. Much further attention should be paid to the biosafety of the novel materials before applied in practice. Meanwhile, both the process and raw materials must be optimized as RFP is a chemotherapeutic agent that has numerous side effects on the liver and kidney after long-term use.

In conclusion, this study provides a novel strategy for treating bone defects caused by TB, which will be a new option in the field of bone reconstruction. Meanwhile, this approach is a versatile method of bio-functionalization that could be developed for use with other orthopedic problems by modulating drug release profiles.
